# Early Events in Xenograft Development from the Human Embryonic Stem Cell Line HS181 - Resemblance with an Initial Multiple Epiblast Formation

**DOI:** 10.1371/journal.pone.0027741

**Published:** 2011-11-30

**Authors:** Karin Gertow, Jessica Cedervall, Seema Jamil, Rouknuddin Ali, Marta P. Imreh, Miklos Gulyas, Bengt Sandstedt, Lars Ährlund-Richter

**Affiliations:** 1 Department of Women's and Children's Health, Karolinska Institutet, Stockholm, Sweden; 2 Department of Clinical Science, Intervention and Technology, Karolinska Institutet, Stockholm, Sweden; 3 Department of Genetics and Pathology, Uppsala University, Uppsala, Sweden; University of Medicine and Dentistry of New Jersey, United States of America

## Abstract

Xenografting is widely used for assessing *in vivo* pluripotency of human stem cell populations. Here, we report on early to late events in the development of mature experimental teratoma from a well-characterized human embryonic stem cell (HESC) line, HS181. The results show an embryonic process, increasingly chaotic. Active proliferation of the stem cell derived cellular progeny was detected already at day 5, and characterized by the appearance of multiple sites of engraftment, with structures of single or pseudostratified columnar epithelium surrounding small cavities. The striking histological resemblance to developing embryonic ectoderm, and the formation of epiblast-like structures was supported by the expression of the markers OCT4, NANOG, SSEA-4 and KLF4, but a lack of REX1. The early neural marker NESTIN was uniformly expressed, while markers linked to gastrulation, such as BMP-4, NODAL or BRACHYURY were not detected. Thus, observations on day 5 indicated differentiation comparable to the most early transient cell populations in human post implantation development. Confirming and expanding on previous findings from HS181 xenografts, these early events were followed by an increasingly chaotic development, incorporated in the formation of a benign teratoma with complex embryonic components. In the mature HS181 teratomas not all types of organs/tissues were detected, indicating a restricted differentiation, and a lack of adequate spatial developmental cues during the further teratoma formation. Uniquely, a kinetic alignment of rare complex structures was made to human embryos at diagnosed gestation stages, showing minor kinetic deviations between HS181 teratoma and the human counterpart.

## Introduction

Studies *in vitro* have revealed that human embryonic stem cells (HESC) can recapitulate key developmental differentiation events and exhibit a remarkable capacity to differentiate into diverse specialized cell types *in vitro* [1]. Furthermore, HESC are known to spontaneously differentiate into derivatives of all germ layers when xenografted *in vivo* [2]. The developing growths from xenografted HESC with normal karyotype show histologic analogies with clinical benign mature teratoma and represent chaotic embryonic tissues and initial organoid development [3]–[5]. Consequently, the “teratoma assay” has become a standard for evaluating *in vivo* pluripotency of human cell lines [6]–[8].

Although by many referred to as tumors, experimental teratomas induced by pluripotent stem cells can alternatively be regarded as a manifestation of a failed progress of embryonic development resulting from the ectopic implantation [9]. It has been hypothesized, as reviewed e.g. by Blum and Benvenisto [3], that *in vivo* differentiation of HESC would resemble normal embryonic processes, albeit in a disorganized manner. Using systematic histological examinations and a large set of antibodies, we have earlier described in detail the various tissues within mature teratoma originating from xenografts of the HESC line HS181 [10]. These observations, extending on previously published literature on *in vivo* development of HESC, illustrated HS181 differentiation into discernible and probably functional tissues and also underscored the relative participation of mouse and human cells.

We are not aware of any published study on *in vivo* kinetics or distribution of tissues from the three germ layers in HESC teratoma. Here, we describe a first attempt, again using the well-characterized HS181 cell line, to study also the early stages of HESC mouse xenografts. Interestingly, the resulting growth showed pronounced similarities to embryonic ectoderm/epiblast formation, compatible, and in time aligned, with the most early transient cell populations in human post implantation development.

## Results

### Events days 5–10

**Figure 1:**
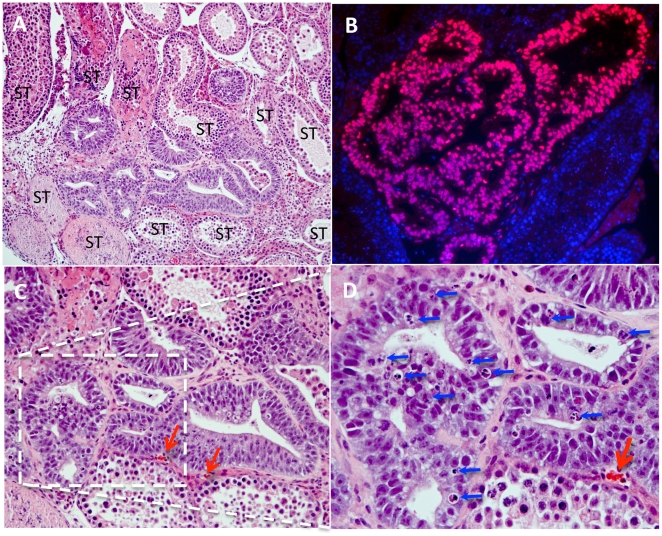
Histology at day 5. (A) HE stained section of mouse testis at day 5 following the injection of HS181 cells. Surrounded by mouse seminiferous tubules ( = ST) a site of initiated growth dominated by multiple appearance of epithelia; in the center of the picture (10x orig. magnification). (B) Illustrates how the human origin of such implanted structures can verified using FISH analysis with human specific probe (40x orig magnification). (C) Illustrates bulging single-and multilayered epithelia, and in (D) higher magnification of the boxed area in C, showing ample mitosis and frequent apoptotic bodies (arrows). Red arrows indicate mouse blood vessels adjacent to implantation (C = 20x and D = 40x orginal magnification).

Three out of five samples (half teratoma; see material and methods) were day 5 positive for human cells and found to include visible sites of HS181 engraftment. In the positive samples, multiple sites of engraftment were found located interstitially between the mouse seminiferous tubules, and consisted of structures characterized by a tubular pattern lined with a single or pseudostratified columnar epithelium (FIG 1A–D). The human origin of these cells was positively verified by FISH analysis using a human specific probe (FIG 1B). Mitotic and apoptotic figures were detected, indicating active proliferation and some cell death.

Also at day 5, human cells with a non-epithelial morphology, possibly representing primary endoderm, were observed adjacent to and surrounding the epithelia formation (FIG 1A–D). In the proximity of the engraftment, in the testis tissues, an increased frequency of mouse vascular structures were detected by day 5 (FIG 1D) and onwards.

**Figure 2:**
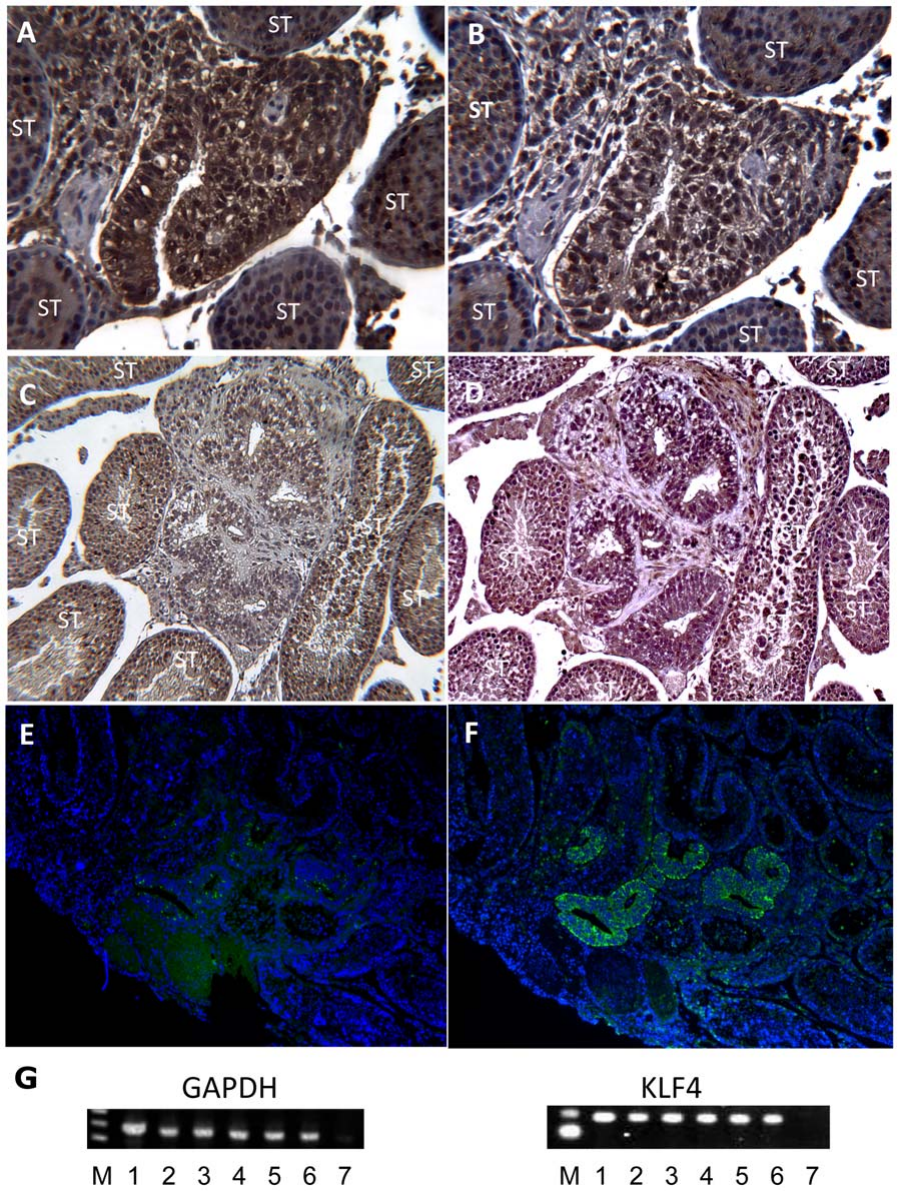
Marker studies day 5. (A) IHC for OCT-4; positive staining of epithelial and non-epithelial structures. (B) IHC for NANOG; positive staining of epithelial and non-epithelial structures. (C) IHC for FGF5; negative. (D) IHC for REX-1; negative. (E) IHC showing positive staining for SSEA-4 in low numbers of cells in epithelia. (F) IHC showing homogenous expression of NESTIN in all epithelia. IHC was in all cases counterstained with DAPI (blue). A–F = 20x original magnification. (G) RT-PCR analysis. Lanes; M = 100 bp ladder. 1 = HS181p38 (positive control). 2–6 = Testis from 5 animals following injections with HS181p38 cells 5 days earlier. 7 = Reaction mix without template (negative control). GAPDH PCR expected product length = 475 bp. KLF4 PCR expected product length = 182 bp.

The presence of embryonic epithelium was further supported by marker analysis. The day 5 epithelial tissue was found positive for markers such as OCT4, NANOG and SSEA-4 (FIG 2A,B,E), but negative for markers FGF5 and REX1 (FIG 2C,D). Notably, the early neural marker NESTIN, was found in all epithelia at this stage (FIG 2F). Also, the human KLF4 gene was expressed (Fig 2G). Transcription of BMP-4, NODAL BRACHYURY or MIXL-1 was not detected using RT-PCR on day 5 samples (data not shown).

Four out of five testes samples at day 10 included signs of engraftment. At this stage a gradual increase of the epithelial multilayered structures was apparent.

### Events days 20–30

Three out of five samples were positive for human cells at day 20 and the same frequency was found for the samples of day 30.

**Figure 3:**
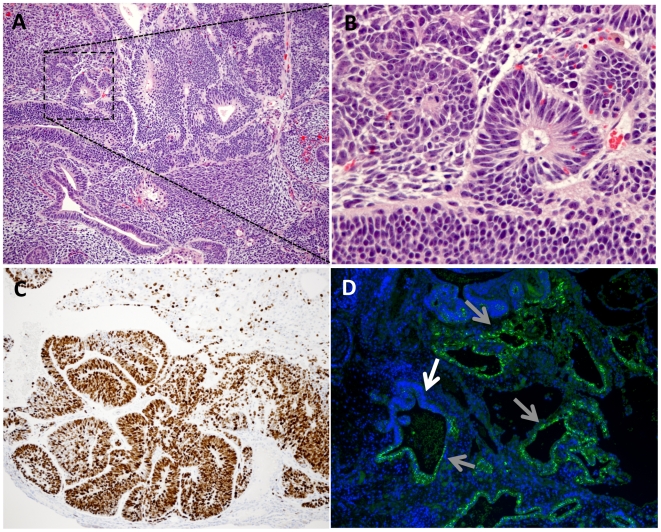
Histology and marker studies day 30. (A) HE staining of teratoma section day 30 showing diverse teratoma tissues. (B) Higher magnification of detail in A (dashed box), demonstrating expanding multilayer epithelium with low frequency or lack of apoptotic bodies. (C) IHC; intense Ki 67 staining in area of epithelial cells indicating high proliferation rates. (D) IHC showing SSEA4 positive cells at multiple sites of single layer epithelium (examplifed by the grey arrows) and a low frequency of SSEA4 cells in multilayered epithelium (white arrow). A = 10x, B = 40x and C,D = 20x original magnification.

Contrary to earlier time points, the occurrence of apoptotic figures was low in the teratoma growth at day 30 (exemplified in FIG 3A,B). Notably, a gradual destruction of the host testicular parenchyma occurred, with the first signs of degeneration of seminiferous tubules day 5, to reaching a near complete destruction of the host tissues at day 30 (not shown).

**Figure 4:**
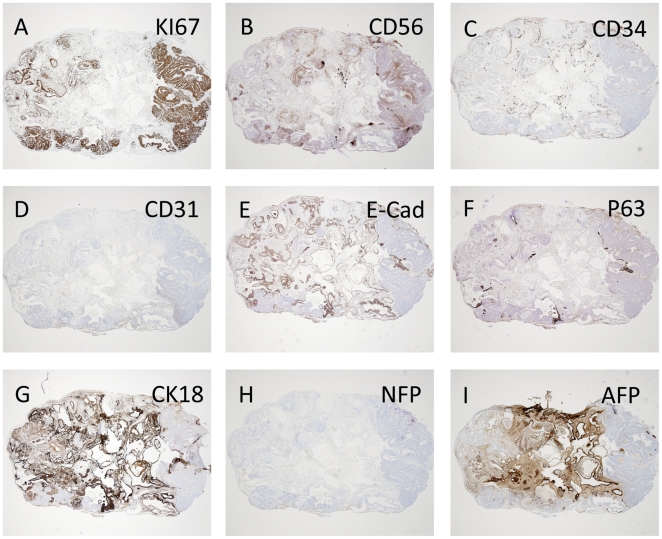
Sections from HS181 day 30 teratoma. (A) IHC; Ki67 staining (mitotic marker). (B) IHC; CD56/NCAM (Neural cell adhesion molecule). (C) IHC: sparse staining of CD34 and (D) IHC; CD31/PECAM (Platelet endothelial cell adhesion molecule). (E) E-CAD (epithelial cadherin). (F) IHC: P63 (epithelial cells). (G) IHC; CK18 (Cytokeratin 18). (H) IHC; Negative staining for NFP (Neurofilament protein). (I) IHC; AFP (alfafetoprotein). A–I = 10x original magnification.

At day 30, diversified teratoma tissues started to occur, dominated by expanding multilayered epithelium (FIG 3A,B, and 4). The pluripotency marker SSEA4 was still detected in single layered epithelia day 30, while multilayered structures at this time point tended to be negative for this marker (FIG 3D). Figure 4A–I depicts serial sections from a day 30 teratoma stained with an array of differentiation markers, illustrating a non homogenous intra-teratoma distribution of proliferative areas (Ki67) largely overlapping with areas stained with CD56 (here a marker for ectoderm) and excluding areas stained with AFP (early marker for endoderm).

High rates of proliferation, indicated by Ki67 staining, could be observed mainly in areas compatible with neuroepithelium (FIG 3C and overview in FIG 4A). From day 20 to day 30 the testis outer diameter was nearly doubled as judged by palpation (data not shown). In line with previous reports on HS181 and also other HESC lines [10], [11], the vascularization was mainly of host origin, with only sparse presence of immature human vessels, indicated by CD31/34 staining (FIG 4C,D).

Also, from this time point and after, cystic components containing gelatinous liquid were frequently detected in the majority of teratomas (FIG 4).

Notably, neither mesoderm structures compatible with somites, nor structures suggesting early heart formation, were found in any of the preparations throughout the study. An *in vitro* potential of HS181 for cardiac development of HS181 cells has however been previously shown by development of beaters [12], [13].

**Figure 5:**
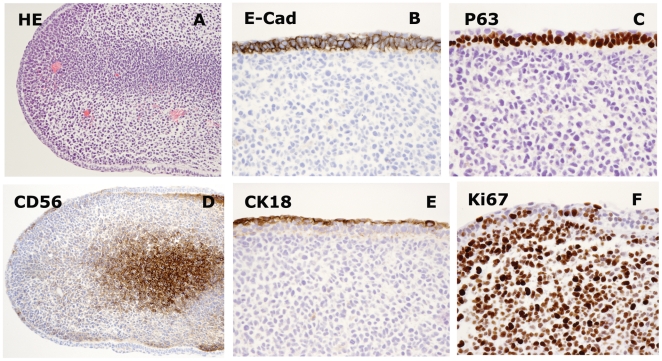
Day 30 development compatible with early limb bud formation. (A) HE staining showing structure resembling limb bud morphology, containing micro vessels. (B) IHC; E-cadherin is found in the ectodermal rim of which the second layer is p63 positive (C), while the most apical cells are CK18 positive (E). Mesenchymal condensation was indicated by CD56 staining (D) and the internal mesenchymal core showed to be highly proliferative by Ki67 staining (F), while the ectodermal surface contained more post proliferative cells. A = 10x and B,C,D,E,F = 40x original magnification.

In one exceptional case at day 30, the teratoma showed a separate structure with features characteristic for early limb formation (FIG 5). In this case, a centrally condensing mesenchyme was covered by a thin, two-layered epithelium, with the peripheral layer consisting of thin elongated cells (FIG 5A). The epithelial cell layers were outlined with E-CADHERIN (FIG 5B), compatible with the situation in early differentiation of the mammalian epidermis. Fully consistent with early epidermal development, IHC revealed intense expression of nuclear P63 (FIG 5C). Peripheral cells were flat and positive for CK18 (FIG 5D). Expression of the neural cell adhesion molecule (N-CAM/CD56), a marker of limb morphogenesis and a finding characteristic of early pre-cartilaginous development, confirmed central mesenchymal condensation (FIG 5E) [14]. Scattered cells localized basally in the epidermis were proliferative, and an intense proliferative activity was also seen throughout the mesenchyme (FIG 5F).

### Events day 60

At day 60, four out of the five testes showed palpable teratoma growth. All teratomas were at day 60 still well encapsulated and expanding rather than infiltrating the testicular capsule. No evidence for the appearance of malignant phenotypes was detected. As expected, a rich variety of tissues, with a chaotic distribution, were observed.

**Figure 6:**
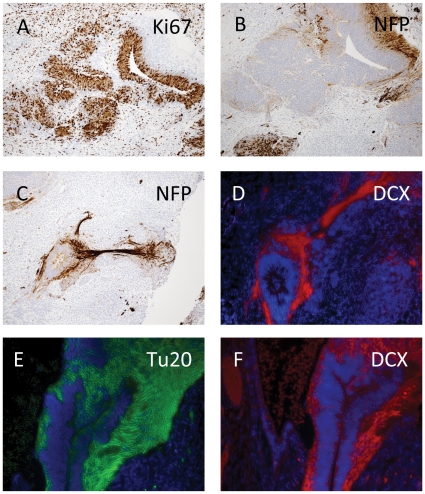
Neural differentiation in mature HS181 teratoma. Day 60 teratoma section with IHC staining for Ki67 (A) indicating proliferative areas of multilayered neuroepithelium, and in a nearby section is stained for NFP (B), this way illustrating the segregation of proliferative and post mitotic zones. Structure compatible with a gangliotic area with nerve fibers, stained for NFP (C) and for doublecortin (DCX) (D). A neuralepithelial structure showing areas of positive staining for βIII-tubulin (TU20) (E) and for doublecortin (F). A–F = 20x original magnification.

Staining for Ki67 indicated that proliferating multilayered neuroepithelium was still found day 60 and composed of both proliferative compartments, as well as post-mitotic neuronal precursors (Fig 6A). Neurogenesis, with presence of ganglionic areas and nerve fibers, was supported by staining with NFP (Fig 6B,C), βIII-tubulin (Fig 6E) and doublecortin (Fig 6D,F). Thus, in this respect markers of the neural maturation from HESC appeared day 60 to match an early stage of cortical plate development.

As predictable from the findings at day 30 of pre-cartilaginous development, day 60 showed a gradient of cartilage maturing toward stages of early endochondral bone formation. Advanced structures compatible with renal development were rare findings at day 60. These structures were found in association with a condensing mesenchyme of nephrogenic character, positive for WT1 staining and with partially vascularized primitive glomeruli and tubuli (data not shown), in line with previous reports [2], [10], [15]–[18].

**Figure 7:**
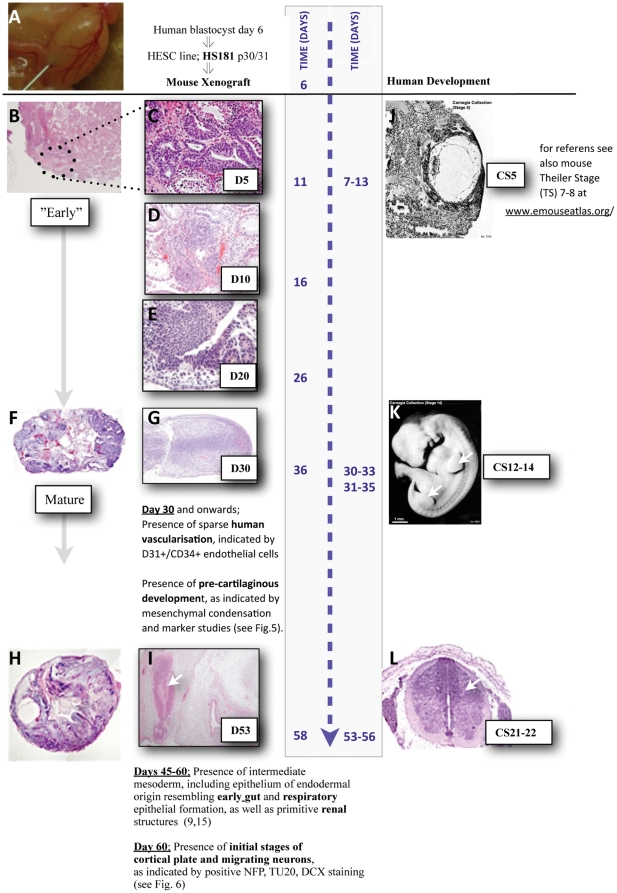
Time alignment with human development. Summary of development in HS181 teratoma (left side), compared to available information on human early development (right side). (A) Illustrates a mouse testes injected with HS181 cells ( = teratoma assay day 0). In (B) multiple initial human structures at day 5 of teratoma assay are indicated (dotted circle). (C) Shows a higher magnification of the epiblast-like epithelial structures (see also Figure 1C). The structures show a striking morphological overlap with mouse Theiler Stages 7/8, best matching the human Carnegie stage (CS) 5 (human gestation days 7–13). Notably, the HS181 cell line originated from a blastocyst on day 6 after fertilization, hence the day 5 of teratoma assay potentially align to day 11 of differentiation post fertilisation. Figures (D) and (E) show the gradual increase of the epithelial multilayered structures at days 10 and 20 (see also Figure 3). (F) Shows an overview of day 30 mature teratoma and (G) a larger magnification of a limb bud like structure (see text and Figure 3). (H) Shows an overview of a HS181/p20 mature teratoma at day 53 and (I) a teratoma structure largely resembling (L) suggesting a ventricular zone with central channel (indicated with an arrow). Figures J–L illustrates available time matched information from human development, (J) Depicts an implantation site at human CS5, and (K) a human embryo at CS14 (arrows indicate upper and lower limb buds). (L) Shows a human embryo at CS21–22, illustrating a ventricular zone with central channel. (Reference picture from the Pathology unit Danderyd Hospital, Sweden.) Illustrations of CS5 and CS14 (J,K) were kindly provided by Dr M. Hill, UNSW.

Figure 7 shows a summary of recognized advanced tissues in HS181 teratoma, time aligned with human development. Teratoma development with striking similarities to human fetal development were revealed (FIG 7J,K,L).

## Discussion

Studies of mice have provided detailed important information on mammal post implantation development (see e.g. The e-Mouse Atlas Project (EMAP): www.emouseatlas.org/emap/). From this, a striking morphological overlap can be appreciated between the mouse Theiler Stages (TS) 7 and 8 and the here observed structures in day 5 HS181 xenografts (Fig 1–2). Parallels between mouse and human developmental stages have been described [19], and suggest that TS8 best matches the human Carnegie stage (CS) 5, equivalent to gestation days 7–13. While considerably less is known about the earliest stages of human post implantation, the HS181 development at day 5 showed resemblance to human post implantation development as it is recognized at human CS5a–c [20]. In analogy, a resemblance could also be perceived with preimplantation stages of baboon as beautifully illustrated by Tarara, et al. [21].

Teratomas induced by pluripotent stem cells have generally been referred to as benign tumor formation [3]. Our results studying the pluripotent HESC line HS181 supports the notion of a failed, increasingly chaotic, embryonic process. Notably, the early HS181 xenografts exhibited multiple sites of growth initiation, in this aspect supporting the suggestion from Blum and Benvenisto that *in vivo* differentiation of HESC is polyclonal [22]. Furthermore, the early structures showed both pseudostratified and multilayered epithelium, suggesting presence of asynchronous developmental stages.

The HESC line HS181 was derived from the inner cell mass of a blastocyst on day 6 after fertilization [12], with an embryo grading score of 4BB [23], i.e. an expanded blastocyst with small cell numbers in the inner cell mass and trophectoderm. In this context, it was intriguing that HS181 early xenografts could reorganize into epithelial structures with a marker profile largely resembling developing embryonic ectoderm. Several findings supported the presence of developing neural ectoderm. Nestin is expressed in the majority of mitotically active neural progenitors and commonly interpreted as a marker for neuronal stem cells [24]. The finding of NESTIN expression in all initial epithelia day 5 thus indicated the presence of primitive neural ectoderm. SSEA-4 [25], a marker linked to stemness, could be detected in single cell epithelia in the teratomas up to day 30. NODAL signaling is essential for the maintenance of HESC pluripotency [26], [27] and is present in undifferentiated HS181 cells [7] (and data not shown). Using sensitive RT-PCR we did not detect expression of NODAL in day 5 teratomas. Nodal is reported essential for primitive streak formation, as well as for early mesoderm and endoderm development in mouse [28], [29]. However, inhibition of Nodal-/Activin-signaling is essential for induction of neuroectoderm *in vitro* and during gastrulation *in vivo* [30], [31]. Thus, the absence of NODAL at day 5 is in line with a presence of primitive neural ectoderm.

NANOG is reported to repress differentiation into primitive endoderm [32] and was found to be expressed day 5. Fibroblast growth factor 5 (FGF5) is a major marker for pluripotent primitive ectoderm just prior to gastrulation [33], [34]. It is also expressed in the undifferentiated HS181 cells ([7], and data not shown). Surprisingly, the day 5 ectoderm-like cells were negative for FGF5 using IHC. Vallier, et al. [26] reported that human equivalents of early primitive ectoderm-like cells (EPL cells, as described by Rathjen, et al. [35]) did not express FGF5. Although different experimental systems were used, the data from Vallier, et al. are of interest in the context of our negative findings for FGF5.

The NESTIN-positive epithelial day 5 structures were BMP4 negative (as shown by RT-PCR in 3/3 samples), a condition likely to have influenced the early neuronal dominance. The first 30 days were almost completely dominated by neuroepithelium.

Besides the early devoid of BMP-4, other markers for gastrulation, e.g. BRACHYURY, SHH, and WNT's were also initially absent. In the mature teratomas, only limited presence of derivatives from the paraxial or lateral mesoderm was observed and a smaller portion of other ectodermal derivatives was identified. In this perspective, it is possible that the extensive cartilage observed could in fact have been derived from the forming of neural crest or prechordal plate mesoderm.

When subjected to neural *in vitro* differentiation protocols, or spontaneously just following suboptimal culture conditions, HS181, like other HESC lines, forms radial arrangements of elongated columnar cells (“neural rosettes”), surrounded by flat epithelial cells. In our experience, for HS181, this occurs approximately day 9 following the removal of conditions supporting undifferentiated growth (unpublished). *In vivo* neural rosette formation in the HS181-xenografts could first be observed from day 10, thus in this respect closely following the *in vitro* findings.

Collectively, the above discussed results suggest that early HESC xenografts may provide a hitherto overlooked route for studies of the most early transient cell populations in development.

For ethical reasons, there is no apparent natural ectopic site for reinstating HESC to *in vivo* development. Site-dependent tissue composition of HESC-teratoma was not detected in studies by Prokhorova, et al. [36], while Cooke, et al. reported a more aggressive character following injections into liver [37]. In our studies, the site of injection was chosen for reasons described earlier [10]; i.e. the testis is not a vital organ, it is relatively easy to access and the growth can at least be partially monitored by external examination. However, the hypoxic status in mouse testis [38], [39] together with an explicit germ cell-promoting environment, as well as species related interference [40], may well have interfered with the engraftment. A gradual destruction of the mouse testicular parenchyma was evident in that some degeneration of seminiferous tubules occurred as early as day 5. This was followed by a nearly complete destruction of the host tissues, leaving only dispersed remaining host cells embedded within human tissues by day 30. Thus, while the relative influence from a human microenvironment increased with the build up of tissues, the influence from the host testis gradually decreased and was in the mature teratoma limited to mainly blood support. We did not at any stage of teratoma formation observe cellular immune infiltration. Immune interactions with the host was minimized, since the host SCID/Beige mice lack both T and B lymphocytes due to a defect in V(D)J recombination, as well as macrophage defects and selectively impaired NK cell functions [41], [42].

Extending on the results of previous studies, areas of more advanced embryonic structures were observed. A unique approach used in this study was the opportunity for direct morphological comparisons to sections from human embryos at diagnosed gestation stages. This included three particularly notable observations. i) In human development, upper extremities appear as early visible structures from CS12 (gestation day 28), followed by the lower extremities emerging at CS14 (gestation days 33–35). Day 30 following HS181 teratoma initiation, a single case of limb bud formation (upper or lower) was observed (Figure 6G). As illustrated in Figures 6 the total time period of *in vitro/vivo* differentiation (36 days; i.e. 6 days of *in vitro* culture, plus 30 days *in vivo* as xenograft) preceding the observed occurrence of the teratoma bud-like structure is thus in agreement with the kinetics of human limb bud development. ii) Similarly, at day 53, we observed a single case of a trachea-like embryonic structure with suggestive respiratory epithelium and surrounding muscularis externa (Figures 6I). iii) The most advanced structure was observed at day 53 and comprised of a single neural tube like structure diligently harmonizing with the histology of a ventricular zone with central channel (Figures 6J). Both these latter embryonic structures could be time matched with reference material from a human embryo at approximately CS21–22, equivalent to gestation days 53–56 (illustrated in Figures 7K and 7L, respectively), indicating less than one week discrepancy of the total time of *in vitro/vivo* differentiation preceding the occurrence of these teratoma structures, compared to human embryonic development.

In our present and previous studies using HS181, we have in mature teratomas (>45 days) observed derivatives of intermediate mesoderm including epithelium of endodermal origin resembling early gut- and respiratory epithelial formation, as well as primitive renal structures [10], [15].

Advanced structures were rare and always occurring within a progressively disorganized context and with apparently random surroundings. Although several major developmental milestones resembling gastrulation, neurulation etc could be observed, others were missing, e.g. formation of somites (expected at CS9) and heart (CS12). The absence of detectable somitogenesis could possibly have contributed to the chaotic progression [43]–[45].

Together, these *in vivo* results demonstrate the truly pluripotent nature of the HS181 cell line (with all three germ layers represented in xenografts), as well as a parallel restricted *in vivo* development. This restriction could possibly partly be caused by an imbalance between inductive and suppressive signals for gastrulation and/or the distribution of the germ layers. Based on studies on other HESC lines and literature reports, we tentatively suggest that the observed strong bias for neuronal development was likely due to restrictions endogenous for the cultured HS181 cells, and that inter-line variations can be expected [7], [46]. Alternatively, the xenograft model *per se* may be the cause of the restricting development. Detailed studies with a sizeable set of different HESC lines are needed to solve this question, as well as to learn about the possible generality of some of our findings using the HS181 line. However, this initial stand-alone study on the HESC line HS181 provides, for the first time, a detailed histology of early teratoma development in parallel to marker analysis on progression of typical events in benign HESC teratoma formation, as well as evidence for an at least in time partly aligned embryonic development.

## Materials and Methods

### Ethical permissions

This study was performed in strict accordance with permission for experiments using human embryonic stem cells, from the Local Ethics Committee at Karolinska Institute (114/00), and for animal experimentation from the regional ethical committee (Stockholms norra djurförsöksetiska nämnd; Dnr S172-03 and N105/07).

### Cell culture

The HESC line HS181, 46XX, p30/31 [12] was cultured as previously described [47]. The cells used for this study were all from the very same batch and concurrently injected, thus reducing differences due to varying quality of the cells. (NB. Figure 7H illustrates a teratoma resulted from a separate injection of HS181 p20).

### Mice

Male C.B.-17/GbmsTac-scid-bgDF N7 mice, age 6 weeks, were obtained from M&B, (Denmark) and kept under isolator conditions in M2 cages on aspen wood chips (Beekay bedding, Scanbur B&K AB, Sweden) with free access to water and rodent diet (RM3(P) irr, Special Diet Services, UK), using artificial light from 6.a.m. to 6 p.m, and room temperature between 24+/−2°C, and humidity of 55%+/−10%.

Groups of 5 animals were used. Testes from non-injected animals were used as controls.

### Injection of HESC, development and analysis of teratoma formation

Implantations were performed as described previously [10]. HS181 cells (passage 30–50, 3–4 days after splitting, i.e. in logarithmic growth phase) were harvested mechanically immediately prior to implantation, and approximately 10^4^ cells were inoculated beneath the testicular capsule after the animals were anesthetized using Isoflurane (Baxter, UK). Following this single HS181 injection, at indicated time points, the testis was cut in two halves using a razor blade, for either histology or RNA analysis.

Engraftment, i.e. presence of HS181 derived cells (teratoma formation), was for each sample (at time points days 5, 10 and 20) assayed by human-specific RT-PCR for GAPDH (data not shown), or by Hematoxylin and Eosin (HE) staining and histology (at time points days 30 and 60). Engraftment was also verified by fluorescent in situ hybridization (FISH), using a human-specific probe (FIG 1C).

In line with previous reports [10], the used number of HS181 cells resulted day 60 in palpable teratomas in 4/5 mice (80%). Evidence for HS181 engraftment was correspondingly observed in 17/40 (42%) of the analyzed “half teratoma” samples.

For the histology/IHC analysis, sectioning with 5 µm sections was used and for practical reasons we limited the analysis to 100 HE stained sections per testis sample. A summary of the observations on engraftment and IHC for each sample analyzed (half teratoma) is presented in Table S1.

### Human specific fluorescent In Situ Hybridization (FISH)

A human specific FISH probe (Spectrum Red labeled total human genomic DNA, Vysis Inc., Downers Grove, IL) was used to verify the human origin of the formed tissues, as previously described [10].

### Immunohistochemistry (IHC)

IHC was used to identify the structures and characterize differences in maturation in the sections obtained from HESC teratomas at the indicated time points. The panel of antibodies used is summarized in Table S2. Sections were deparaffinized in xylene and rehydrated through graded series of ethanol. Highlighted markers (*) in Table S2 were performed on TechMate TM 500 Plus equipment, using the ChemMate Detection Kit and Peroxidase/DAB (all from DAKO Cytomation, Glostrup, Denmark), and counterstained with hematoxylin.

Sections analyzed with antibodies against NESTIN, βIII-Tubulin, doublecortin and SSEA-4 were blocked with 3% BSA (fraction V, Sigma-Aldrich, St. Louis, MO) and 0.1% BSA-c (Aurion, 900.022) for 40 min at room temperature. After blocking, the sections were incubated with primary antibodies for 1 hour at room temperature. Following the incubation with the primary antibody, the slides were washed with PBS and incubated with a secondary antibody, AlexaFluor488 (Molecular Probes Inc. Eugene, OR), for 40 min at room temperature. After washing with PBS, the sections were mounted with Vectashield containing DAPI (Vector Laboratories Inc., Burlingame, CA). Stainings for Oct4, Nanog, Rex1, FGF5 and Brachyury were performed using Vectastain ABC Kit (Vector Laboratories Inc. Burlingame, CA) or Goat HRP-Polymer Kit (Biocare Medical, Concord, CA), depending on origin of the primary antibody (see Table S2). The procedure recommended by the manufacturer was followed. Control experiments were performed using unspecific isotypic antibodies.

### Reverse transcriptase - polymerase chain reaction (RT-PCR)

Total RNA was extracted from the tissues using TRIzol reagent following the manufacturer's protocol (Invitrogen, Carlsbad, CA). Thereafter, the RNA was DNase-treated (Invitrogen, Carlsbad, CA) to avoid DNA contamination, precipitated and resolved in DEPC treated water to obtain final concentration of 0.1 µg/µl. Complementary DNA was synthesized using Superscript III First Strand Synthesis System (Invitrogen, Carlsbad, CA) according to the recommended protocol. Platinum Taq-polymerase was used in the following PCR reaction containing cDNA synthesized from 20 ng total RNA. The qualities of the individual RNA preparations were corroborated by a positive amplification of β-actin or GAPDH. Markers studied and primer sequences used are indicated in Table S3.

## Supporting Information

Table S1 Summary of observations on engraftment and results on IHC for each sample (half teratoma*) analysed. * Following injections of HS181 cells, at indicated time points, the testis was cut in two halves using a razor blade and one half used for histology/IHC. ** Engraftment, i.e. presence of HS181 derived cells (teratoma formation), was for each sample assayed by human-specific RT-PCR for GAPDH, or human specific fluorescent in situ hybridization (FISH), or by HE staining and histology (palpable teratomas). Samples found positive for teratoma are indicated by grey shade in the table.
(DOC)

Table S2 **Immunohistochemistry: Primary antibodies used.** * Staining made on a TechMate TM 500Plus; **†, ††** Staining was performed manually as described in the material and methods section.
(DOC)

Table S3 **RT-PCR; primer sequences and conditions.**
(DOC)
