# Supplementary material for: Early Events in Xenograft Development from the Human Embryonic Stem Cell Line HS181 - Resemblance with an Initial Multiple Epiblast Formation
Source: PLoS One. 2011 Nov 30;6(11):e27741. doi: 10.1371/journal.pone.0027741 (PMC3227586; doi:10.1371/journal.pone.0027741)
Supplement: Table S1 — Summary of observations on engraftment and results on IHC for each sample (half teratoma*) analysed. * Following injections of HS181 cells, at indicated time points, the testis was cut in two halves using a razor blade and one half used for histology/IHC. ** Engraftment, i.e. presence of HS181 derived cells (teratoma formation), was for each sample assayed by human-specific RT-PCR for GAPDH, or human specific fluorescent in situ hybridization (FISH), or by HE staining and histology (palpable teratomas). Samples found positive for teratoma are indicated by grey shade in the table. (DOC) [file pone.0027741.s001.doc]

Table S1: Summary of observations on engraftment and results on IHC for each sample (half teratoma*) analysed.

| **Day 5 series A** | **#5:1** | **#5:2** | **5:3** | **#5:4** | **#5:5** |
| --- | --- | --- | --- | --- | --- |
| Teratoma** | - | + | + | + | - |
| Nestin |  | + |  |  |  |
| SSEA-4 |  | + |  |  |  |

| **Day 5 series B** | **#5:6** | **#5:7** | **#5:8** | **#5:9** | **#5:10** |
| --- | --- | --- | --- | --- | --- |
| Teratoma** | + | **-** | **-** | **-** | **-** |
| Nestin | + |  |  |  |  |
| SSEA-4 | + |  |  |  |  |
| OCT-4 | + |  |  |  |  |
| NANOG | + |  |  |  |  |
| REX1 | + |  |  |  |  |
| FGF5 | - |  |  |  |  |

| **Day 10** | **#10:1** | **#10:2** | **#10:3** | **#10:4** | **#10:5** |
| --- | --- | --- | --- | --- | --- |
| Teratoma** | + | + | + | + | - |
| Nestin | + |  |  |  |  |

| **Day 20** | **#20:1** | **#20:2** | **#20:3** | **#20:4** | **#20:5** |
| --- | --- | --- | --- | --- | --- |
| Teratoma | + | **+** | **+** | **-** | **-** |
| Nestin | + |  |  |  |  |

| **Day 30** | **#30:1** | **#30:2** | **#30:3** | **#30:4** | **#30:5** |
| --- | --- | --- | --- | --- | --- |
| Teratoma* | + | **-** | **-** | **+** | **+** |
| Ki67 |  |  |  | + | + |
| SSEA-4 |  |  |  | + | + |
| CD56 |  |  |  | + | + |
| NFP |  |  |  | - | - |
| E-cadherin |  |  |  | + | + |
| P63 |  |  |  | + | + |
| CK18 |  |  |  | + | + |
| CD34 |  |  |  | + | + |
| CD31 |  |  |  | + | + |
| WT-1 |  |  |  | + | + |
| AFP |  |  |  | + | + |

| **Day 60** | **#60:1** | **#60:2** | **#60:3** | **#60:4** | **#60:5** |
| --- | --- | --- | --- | --- | --- |
| Teratoma** | - | **+** | **-** | **+** | **+** |
| Ki67 |  |  |  | + | + |
| CD56 |  |  |  | + | + |
| Doublecortin |  |  |  | + | + |
| βIII-tubulin |  |  |  | + | + |
| NFP |  |  |  | + | + |
| E-cadherin |  |  |  | + | + |
| P63 |  |  |  | + | + |
| CK18 |  |  |  | + | + |
| CD34 |  |  |  | + | + |
| CD31 |  |  |  | + | + |
| WT-1 |  |  |  | + | + |
