# Supplementary material for: Early Events in Xenograft Development from the Human Embryonic Stem Cell Line HS181 - Resemblance with an Initial Multiple Epiblast Formation
Source: PLoS One. 2011 Nov 30;6(11):e27741. doi: 10.1371/journal.pone.0027741 (PMC3227586; doi:10.1371/journal.pone.0027741)
Supplement: Table S2 — Immunohistochemistry: Primary antibodies used. * Staining made on a TechMate TM 500Plus; †, †† Staining was performed manually as described in the material and methods section. (DOC) [file pone.0027741.s002.doc]

**Table S2: Immunohistochemistry: Primary antibodies used.**

| **Antigen** | **Marker for** | **Dilution** | **Origin** | **Source** |
| --- | --- | --- | --- | --- |
| Ki67 * | Mitotic cells, Proliferation, | 1:200 | Mouse | DAKO Cytomation, Glostrup, Denmark |
| SSEA-4 † | Stage-specific embryonic antigen 4. Stem cell marker | 1:100 | Mouse | Chemicon, CA |
| NESTIN † | Neural stem cell ntermediate filament | 1:200 | Rabbit | Chemicon, CA |
| βIII-Tubulin† | Neuron specific | 1:200 | Mouse | Chemicon, CA |
| Doublecortin† | Marker of migrating neurons | 1:50 | Mouse | Santa Cruz, CA |
| NFP * | Neurofilament protein | 1:50 | Mouse | DAKO Cytomation, Glostrup, Denmark |
| CD56 * | neural cell adhesion molecule (N-CAM) broad adhesion marker | 1:50 | Mouse | NovoCastra Labs, UK |
| E-Cadherin* | Epithelial cadherin. Cell-cell adherence | 1:50 | Mouse | DAKO Cytomation, Glostrup, Denmark |
| P63* | Epithelial cells | 1:100 | Mouse | NeoMarker, CA |
| CK18* | Cytokeratin 18. Simple epithelia | 1:100 | Mouse | DAKO Cytomation, Glostrup, Denmark |
| CD31 * | PECAM-1.  Mature endothelial cells | 1:100 | Mouse | DAKO Cytomation, Glostrup, Denmark |
| CD34 * | Immature endothelial cells | 1:100 | Mouse | DAKO Cytomation, Glostrup, Denmark |
| WT1* | Wilm´s Tumor-1. Transcription factor in kidney development | 1:200 | Mouse | DAKO Cytomation, Glostrup, Denmark |
| FGF5**††** | Expressed in early embryonic development, epiblast | 1:100 | Goat | R&D Systems, MN |
| Rex1**††** | Transcription factor, early development | 1:50 | Rabbit | Nordic Biosite, Sweden |
| Oct4**††** | Transcription factor, early development | 1:50 | Mouse | Chemicon; CA |
| Nanog**††** | Transcription factor, early development | 1:50 | Goat | R&D Systems, MN |
| Brachyury**††** | Expressed in early mesoderm formation | 1:200 | Goat | Santa Cruz Biotech, CA |
