# Supplementary material for: Early Events in Xenograft Development from the Human Embryonic Stem Cell Line HS181 - Resemblance with an Initial Multiple Epiblast Formation
Source: PLoS One. 2011 Nov 30;6(11):e27741. doi: 10.1371/journal.pone.0027741 (PMC3227586; doi:10.1371/journal.pone.0027741)
Supplement: Table S3 — RT-PCR; primer sequences and conditions. (DOC) [file pone.0027741.s003.doc]

**Table S3: RT-PCR; primer sequences and conditions.**

| **Gene** | **Primer sequences** | **Size** (**bp)** | **Annealing**  **Temp.**°**C** | **mM** (**MgCl_2_]** |
| --- | --- | --- | --- | --- |
| **β-actin** | **F:5´-GACATTAAGGAGAAGCRGTGCTATGTT-3´**  **R:5´-GCCTAGAAGCATTTGCGGTGGACGA-3´** | **497** | **58** | **3,0** |
| **NANOG** | **F:5´-CGGCTTCCTCCTCTTCCTCTATAC-3´**  **R:5´-ATCGATTTCACTCATCTTCACACGTC-3´** | **960** | **57** | **1,5** |
| **Klf4** | **F:5´-CTGCGGCAAAACCTACACAA -3´**  **R:5´-GGTCGCATTTTTGGCACTG -3´** | **182** | **51** | **3,0** |
| **Nestin** | **F:5´-CAGCTGGCGCACCTCAAGATG****-3´**  **R:5´-AGGGAAGTTGGGCTCAGGACTGG-3´** | **208** | **49** | **2,0** |
| **Shh** | **F:5´-GAAAGCAGAGAACTCGGTGG-3´**  **R:5´-GGAAAGTGAGGAAGTCGCTG-3´** | **170** | **51** | **5,0** |
| **Brachyury** | **F:5´-GTGACCAAGAACGGCAGGAGG-3´**  **R:5´-TGTTCCGATAGCATAGGGGC -3´** | **706** | **52** | **1,0** |
| **Wnt3** | **F:5´-ACTTCGGCGTGTTAGTGTCC-3´**  **R:5´-ATTTTTCCTTCCGCTTCTCC-3´** | **501** | **51** | **1,0** |
| **Wnt5a** | **F:5´-CAGTTCAAGACCGTGCAGAC-3´**  **R:5´-TGGAACCTACCCATCCCATA-3´** | **501** | **51** | **1,0** |
| **BMP-4** | **F:5´-GCTGAAGTCCACATAGAGCGAGTG-3´**  **R:5´-ACTTCGAGGCGACACTTCTGC-3´** | **785** | **53** | **1,0** |
| **Nodal** | **F:5´-CATGAAAGCTATAGGTGACTTCAT-3´**  **R:5´-TGTAAATGAAGGGCTCAGTGGA-3´** | **250** | **56** | **1,0** |
| **MyoD** | **F:5´-GGG AAG AGT GCG GCG GTG TCG AG-3´**  **R:5´-TCCCAGAAGGGTGCTGCGTGGAA-3´** | **445** | **57** | **1,0** |
